# Identification of TMEM106B amyloid fibrils provides an updated view of TMEM106B biology in health and disease

**DOI:** 10.1007/s00401-022-02486-5

**Published:** 2022-09-02

**Authors:** Jolien Perneel, Rosa Rademakers

**Affiliations:** 1 VIB Center for Molecular Neurology, University of Antwerp, Universiteitsplein 1, Wilrijk, 2610 Antwerp, Belgium; 2 Department of Biomedical Sciences, University of Antwerp, Antwerp, Belgium

**Keywords:** TMEM106B, Amyloid fibrils, Aggregation, Lysosomal dysfunction, Progranulin, Frontotemporal dementia

## Abstract

Since the initial identification of *TMEM106B* as a risk factor for frontotemporal lobar degeneration (FTLD), multiple genetic studies have found *TMEM106B* variants to modulate disease risk in a variety of brain disorders and healthy aging. Neurodegenerative disorders are typically characterized by inclusions of misfolded proteins and since lysosomes are an important site for cellular debris clearance, lysosomal dysfunction has been closely linked to neurodegeneration. Consequently, many causal mutations or genetic risk variants implicated in neurodegenerative diseases encode proteins involved in endosomal–lysosomal function. As an integral lysosomal transmembrane protein, TMEM106B regulates several aspects of lysosomal function and multiple studies have shown that proper TMEM106B protein levels are crucial for maintaining lysosomal health. Yet, the precise function of TMEM106B at the lysosomal membrane is undetermined and it remains unclear how TMEM106B modulates disease risk. Unexpectedly, several independent groups recently showed that the C-terminal domain (AA120-254) of TMEM106B forms amyloid fibrils in the brain of patients with a diverse set of neurodegenerative conditions. The recognition that TMEM106B can form amyloid fibrils and is present across neurodegenerative diseases sheds new light on TMEM106B as a central player in neurodegeneration and brain health, but also raises important new questions. In this review, we summarize current knowledge and place a decade’s worth of TMEM106B research into an exciting new perspective.

## Introduction

Neurodegenerative disorders are characterized by misfolding and aggregation of proteins such as tau, amyloid-β (Aβ), α-synuclein, and TDP-43. These disorders are commonly referred to as proteinopathies and are named more specifically after the aggregating protein, e.g., tauopathies, synucleinopathies, and TDP-43opathies [12, 65]. Filamentous aggregates and inclusions in neuronal and glial cell types will ultimately lead to toxicity and cell death resulting in brain atrophy. Depending on the implicated brain region(s), the degeneration may disrupt core human characteristics such as memory, speech, behavior, personality, and movement. Improving our understanding of disease pathomechanisms that underlie the formation of aggregates as well as detailed knowledge of the structure of filamentous aggregates may offer disease insight and are crucial to aid in both biomarker and therapy development. Recent advances in cryogenic electron microscopy (cryo-EM) have enabled researchers to identify the structure of fibrils extracted from postmortem brain tissue, and over the past years the structure of pathological forms of filaments formed by tau (reviewed in [60]), amyloid-β [31], α-synuclein [58], and TDP-43 [2] have been determined. Several independent cryo-EM groups now report amyloid fibrils in brain tissue of a diverse set of neurodegenerative disorders as well as older neurologically normal individuals to comprise the C-terminal domain (AA120-254/274) of transmembrane protein 106B (TMEM106B), a protein previously shown to modulate disease risk in neurodegeneration and implicated in healthy aging [10, 17, 27, 57].

The identification of TMEM106B amyloid fibrils in postmortem brain tissue offers a new perspective on the involvement of TMEM106B in neurodegeneration and brain health. Here, we review these findings as well as summarize the current understanding of TMEM106B biology and function in both health and disease. Finally, we discuss and speculate on the implications of TMEM106B amyloid fibrils on disease and highlight the potential for biomarker development and therapeutic approaches related to TMEM106B.

### TMEM106B in health and disease

Frontotemporal lobar degeneration (FTLD) is a group of heterogeneous, progressive neurodegenerative disorders representing 10–20% of all dementias with an early disease onset, making FTLD the second most common dementia in people under the age of 65 years [48, 72]. Pathologically, TAR DNA-binding protein 43 (TDP-43) is the most commonly aggregated protein (~ 50% of all cases) found in the brain of FTLD patients (FTLD-TDP), where TDP-43 forms hyperphosphorylated, ubiquitinated inclusions [24, 39]. According to the morphology and distribution of the TDP-43 inclusions, FTLD-TDP is further classified into types A through E [42]. In 2010, *TMEM106B* was identified as a risk-associated gene for FTLD-TDP, where the disease-modulating effect was especially prominent in FTLD-TDP patients harboring pathogenic mutations in the progranulin (*GRN*) gene [14]. Heterozygous loss-of-function mutations in *GRN* (resulting in a 50% loss of progranulin (PGRN) protein) represent ~ 20–25% of cases of FTLD-TDP, yet individuals with *GRN* mutations who also carry a *TMEM106B* ‘protective’ haplotype have approximately 50% lower odds of developing FTLD symptoms [51]. In later studies, the risk-modulating effect was also extended to patients carrying the *C9orf72* hexanucleotide GGGGCC repeat expansion [4, 23].

Since then, multiple studies have established genetic variants in *TMEM106B* as important modifiers of disease risk in a variety of neurodegenerative disorders including other TDP-43 proteinopathies as well as tauopathies, reviewed in [47] and [19]. Moreover, TMEM106B’s risk-modifying capabilities go beyond disease protection alone, as it has been linked to brain aging even in the absence of known brain disease [54]. In addition, TMEM106B has been associated with neuronal proportion, conferring neuronal protection against general aging [38]. The importance of TMEM106B in brain health is further highlighted by its association with cognition [36, 52, 54, 66, 67, 70, 71] and the recent associations with mood disorders such as depression [13, 16, 45].

### TMEM106B genetic haplotypes

In and around *TMEM106B* on chromosome 7p21, several single nucleotide polymorphisms (SNPs) were identified in high linkage disequilibrium, resulting in two common *TMEM106B* haplotypes in the human population [14]. Since it is not currently known which variant on the haplotype is functionally responsible for modulating disease risk, they are collectively referred to as either the risk or protective haplotype. It is the most frequent of these two haplotypes which has consistently been associated with an increased risk for neurodegenerative diseases and poor brain health. However, the functional effect of the risk haplotype, as well as the responsible disease-modifying variant, has been a topic of active discussion.

### TMEM106B structure and function

#### Structure and interactions

**Fig. 1:**
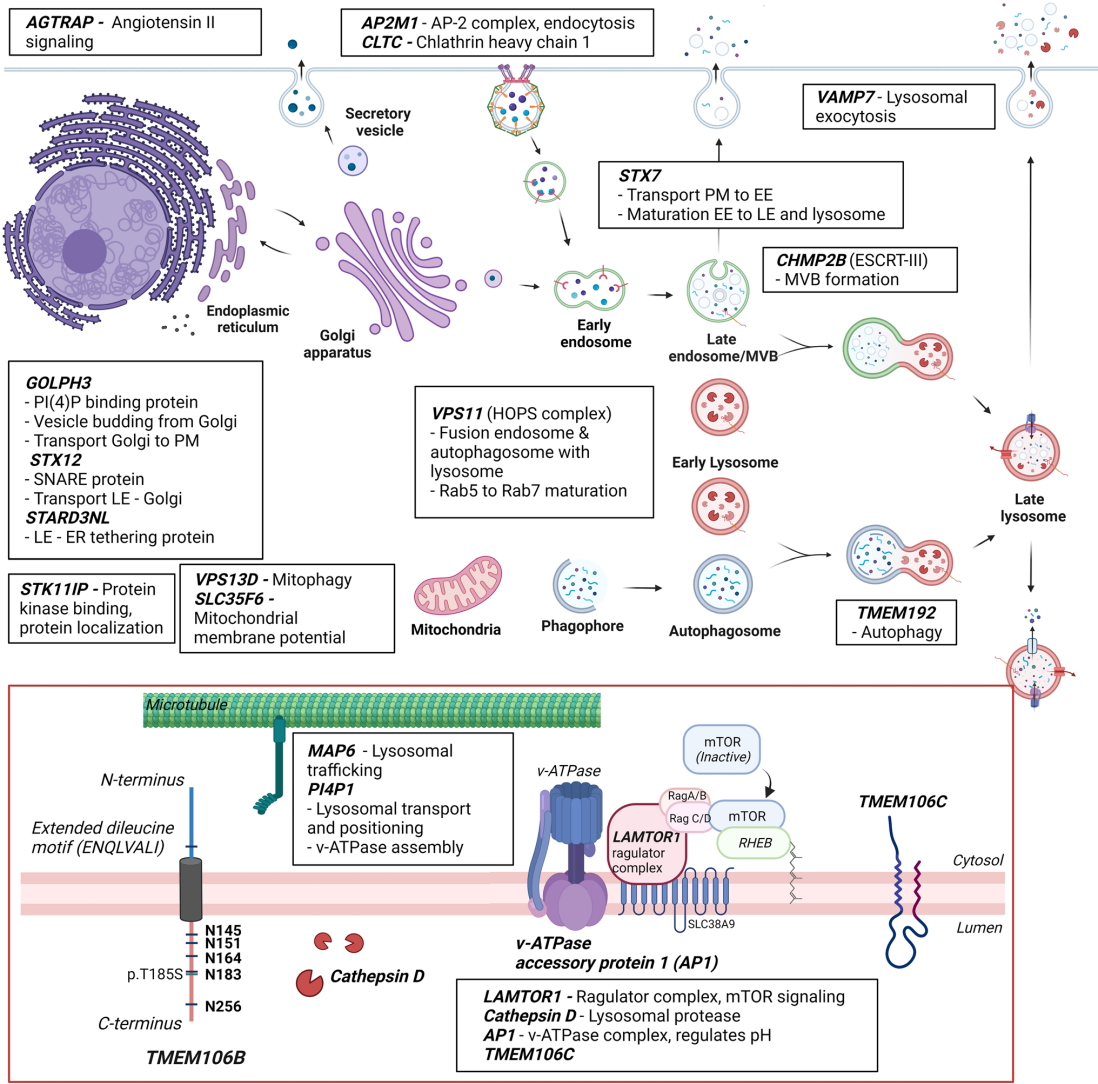
Schematic presentation of reported interaction partners of TMEM106B. All interaction partners are listed in a black box with a short functional description near the respective implicated cellular pathways. A detailed schematic of the lysosomal membrane is depicted in the red box. PM, plasma membrane; ER, endoplasmic reticulum; EE, early endosome; LE, late endosome; MVB, multivesicular body

TMEM106B is a highly glycosylated, single-pass, type II transmembrane protein comprising a total of 274 amino acids. It is localized in the membrane of late endolysosomal compartments, with the N-terminus in the cytosol and the C-terminus within the lumen [34]. The cytoplasmic N-terminus (residues 1–96) is intrinsically disordered without a well-defined secondary or tertiary structure which may offer the ability to dynamically interact with diverse binding partners [29, 37]. A limited number of interaction partners have been reported for TMEM106B [21, 28, 30, 59, 63] (https://opencell.czbiohub.org/target/CID002001), which are schematically presented in Fig. 1. Additionally, TMEM106B can form homo- and heterodimers with its homolog TMEM106C, through a CxxCxGxG motif that is capable of forming a zinc-binding site. The interaction with TMEM106C has been confirmed and the presence of dimers was also observed on western blot; however, the functional importance of the dimers is unknown [37, 63]. The lysosomal sorting of TMEM106B is mediated by an extended dileucine signal located in the N-terminal region (**E**NQ**L**VA**LI**), and abrogation of this signal leads to a diffuse cytosolic distribution of TMEM106B [9].

Following the transmembrane domain (residues 97–117), the C-terminal domain (residues 118–274) resides within the lumen and contains five N–X–T/S glycosylation motifs at N145, N151, N164, N183, and N256. The first three glycosylation sites are reported to result in simple N-glycosylation, while the last two sites are reported to result in complex N-glycosylation. The transport of TMEM106B to the lysosomes is also modulated by its post-translational modifications and depends on its fourth and fifth N-glycosylation sites. The loss of the N183 or N256 complex glycans was shown to result in impaired transport to the endosomes and lysosomes. In these instances, TMEM106B was retained in the endoplasmic reticulum (ER) or mislocalized to the plasma membrane instead [9, 34, 64].

#### Proteolytic processing

Importantly, TMEM106B is proteolytically processed by an unknown protease, most likely a lysosomal protease, to release its C-terminal domain in the lysosomal lumen. This process generates a residual N-terminal fragment (NTF) anchored to the lysosomal membrane. The remaining NTF is further cleaved by signal peptide peptidase-like 2A (SPPL2A), a GxGD aspartyl protease, by intramembrane proteolysis releasing an intracellular domain (ICD) in the cytosol as well as a small C-domain into the lumen [6]. TMEM106B processing likely occurs to modulate its levels on the lysosomal membrane to regulate its function, but a separate function for the ICD or luminal domain cannot be excluded. Many questions remain on (i) which protease(s) is responsible for cleaving the luminal domain, (ii) the precise cleavage site, and (iii) the relevance of the generated peptides beyond the degradation of full-length TMEM106B. The study of this process has been hampered by the lack of antibodies recognizing the luminal domain and the assumption that, at least under normal conditions, the luminal domain would be degraded by the lysosome along with other lysosomal content.

#### Lysosomal function

TMEM106B plays an important role in lysosome function which is demonstrated by the observation that both knock-down and overexpression of TMEM106B affect lysosomal morphology, pH, maturation, trafficking, and exocytosis. Aberrant changes in TMEM106B levels lead to an accumulation of enlarged lysosomes in the perinuclear region which ultimately induces cytotoxicity. In the lysosome, TMEM106B interacts with several partners (Fig. 1) that are critical for proper lysosome formation including CHMP2B (another FTLD-related protein), which is part of the ESCRT-III complex, with v-ATPase, which is crucial for lysosome acidification and with cathepsin D, a lysosomal enzyme [5, 19, 21, 33, 41, 59, 63]. TMEM106B also functionally interacts with MAP6 to control lysosomal trafficking [59]. Overexpression of TMEM106B causes translocation of transcription factor EB (TFEB) to the nucleus and thus induces the upregulation of the coordinated lysosomal expression and regulation (CLEAR) network. The CLEAR network regulates genes involved in lysosomal function and autophagy, identifying TMEM106B as a critical regulator of lysosomal function [33, 63].

### Functional effect of the TMEM106B haplotypes

#### Variants on the TMEM106B haplotype alter TMEM106B levels

Available experimental evidence suggests that variants on the *TMEM106B* haplotypes exert their effect by altering TMEM106B expression, where an increased expression correlates with the risk haplotype. First, the levels of TMEM106B mRNA and protein were significantly increased in *GRN* mutation carriers [8, 11]. Second, the A-allele of a non-coding variant (rs1990620) located on the *TMEM106B* risk haplotype was shown to preferentially recruit the chromatin-organizing protein CCTC-binding factor (CTCF), modulating TMEM106B expression through transcriptional activation due to altered long-range chromatin-looping interactions [22]. Third, also within the haplotype block, there is one coding variant (rs3173615) encoding a threonine to serine change at amino acid position 185 (p.T185S) located in the fourth N–X–T/S glycosylation motif, which has been suggested to contribute to the disease-modifying effect. In vitro, it was shown that TMEM106B carrying the risk allele (T185) had higher expression levels than the protective allele (S185), potentially due to differences in glycosylation at N183 affecting the protein stability and degradation rate [46]. However, another study observed no such effect [5].

The hypothesis that increased levels of TMEM106B may drive the disease-modifying effect was further supported by the observation that *TMEM106B* contains miRNA-132 and miRNA-212 binding sites in its 3′ UTR, which inhibit *TMEM106B* expression upon binding [11]. In neurodegeneration (including Alzheimer’s disease (AD), FTLD-TDP, etc.) the expression of the microRNA132/212 cluster is decreased [18, 25, 50, 56], suggesting an upregulation of TMEM106B expression in disease.

#### Variants on the TMEM106B haplotype alter TMEM106B biology and function

Alternatively, it was suggested that the coding p.T185S variant might influence disease risk irrespective of TMEM106B levels by altering either TMEM106B biology (cleavage, dimerization, etc.) or by affecting binding (or binding affinity) to interaction partners which could both lead to lysosomal dysfunction. Jun and colleagues showed an enhanced binding of the T185 variant as compared to the S185 variant to CHMP2B, especially with its mutant form, which led to a decrease in autophagic flux [28].

### Identification of TMEM106B Fibrils

#### Diseases

**Fig. 2:**
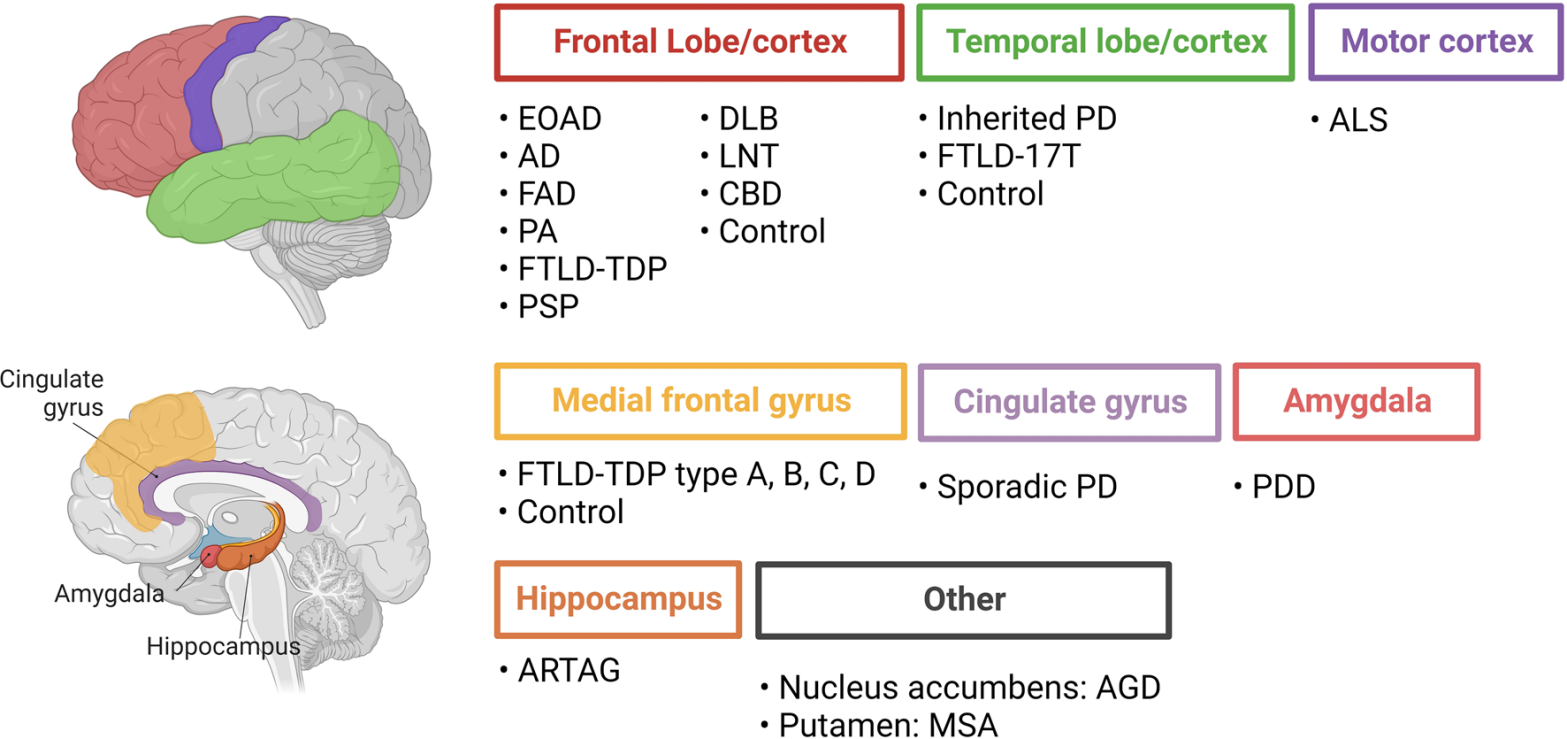
Overview of brain material used in the cryo-EM reports which identified TMEM106B fibrils, including brain region and disease status

Over the past months, several research groups have reported the cryo-EM structures of TMEM106B filaments derived from the brains of a variety of neurodegenerative diseases as well as older neurologically normal individuals [10, 17, 27, 57]. The cryo-EM reports included fibrils obtained from sarkosyl-insoluble fractions of postmortem tissue of individuals with Alzheimer’s disease (AD), argyrophilic grain disease (AGD), amyotrophic lateral sclerosis (ALS), aging-related tau astrogliopathy (ARTAG), progressive supranuclear palsy (PSP), corticobasal degeneration (CBD), dementia with Lewy bodies (DLB), early-onset Alzheimer’s disease (EOAD), sporadic and inherited Parkinson’s disease (PD), PD dementia (PDD), inherited and sporadic frontotemporal lobar degeneration with TDP-43 inclusions (FTLD-TDP) type A, B, C, D, familial frontotemporal dementia and parkinsonism linked to chromosome 17 (FTDP-17T), limbic-predominant neuronal inclusion body 4R tauopathy (LNT), multiple system atrophy (MSA), pathological aging (PA), as well as neurologically normal controls (Fig. 2).

#### Cryo-EM

Although all groups used sarkosyl to extract the fibrils, there was some variability in the fractionation protocol with variations in the stage of sarkosyl addition, the use of ultracentrifugation or low-speed centrifugation, and heating or pronase treatment of the extract. Nevertheless, all reports observed amyloid fibrils with an ordered core comprising residues S120-G254 of TMEM106B. Interestingly, the identification of TMEM106B was distinct between groups. Whereas Chang et al. [10] complemented the cryo-EM with mass spectrometry to identify TMEM106B peptides present in the sarkosyl-insoluble fraction, Jiang et al. [27] modeled two query sequences based on the cryo-EM densities, and Schweighauser and colleagues [57] found their way to TMEM106B based on the fibril’s distinctive glycosylation pattern.

Despite that TDP-43 filaments have been identified in ALS/FTD [2], none of the current studies identified fibrils constituted of TDP-43. Only Jiang and colleagues report abundant non-filamentous aggregates of TDP-43 in FTLD-TDP extracts [27]. Considering Arseni and colleagues [2] used a different extraction method than the current studies, it is plausible that the TDP-43 filaments were lost during the extraction process and reside within another fraction that was not analyzed in the present studies. This emphasizes the importance of the sample preparation protocol in cryo-EM studies and indicates that extraction methods need to be carefully compared, as relatively small changes may influence the purified content. Moreover, the isolation of TDP-43 filaments in future cryo-EM studies aiming to resolve TDP-43 aggregates might require a different approach.

#### TMEM106B fibril structure, polymorphisms, and ultrastructural polymorphs

**Fig. 3:**
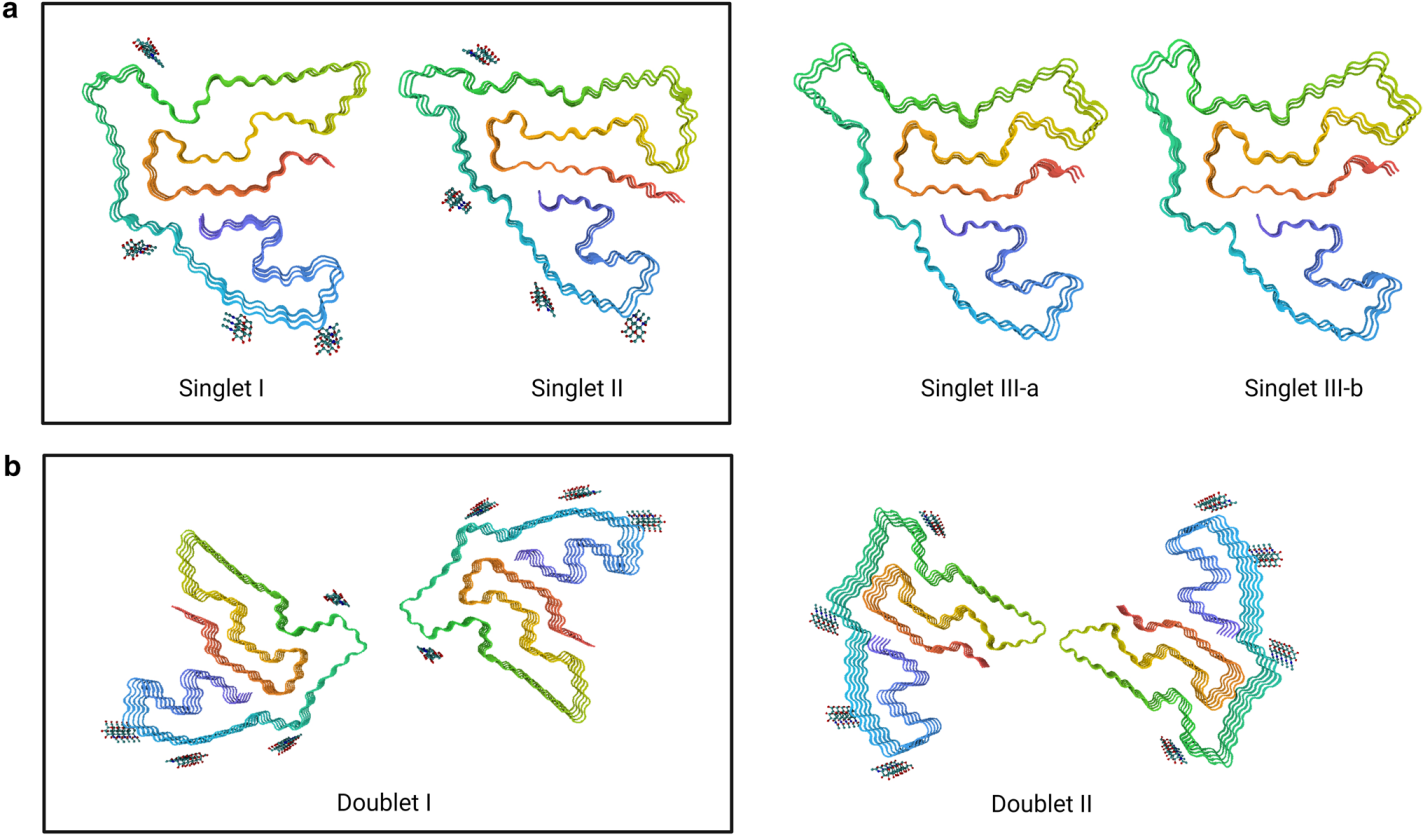
Schematic representation of the distinct forms of TMEM106B fibrils identified in the cryo-EM reports. **a** Singlet polymorphisms and **b** doublet polymorphisms. Boxed singlets and doublet polymorphisms represent the predominant polymorphism(s) identified in multiple reports and/or several disorders. Fibril structures were compared in PDB and extracted from PDB entries 7U16, 7U17, 7QWL, 7QWG, 7SAS, and 7SAR

The TMEM106B fibrils comprise either a single protofilament forming rod-like structures or a doublet formed by two protofilaments forming a twisted ribbon. The structure of several polymorphisms of the protofilaments, four singlet polymorphisms and two doublet polymorphisms, have been reported (Fig. 3). Unlike other amyloid fibrils, no clear relationship between the different polymorphisms and disease status was observed. All polymorphisms share a similar five-layered ordered core consisting of 17–19 β-strands with a highly conserved N-terminal region. All cryo-EM studies report TMEM106B to be fully glycosylated in all folds at the glycosylation sites present within the fibrillar structure (N145, N151, N164, and N183), and the presence of a disulfide bond between C214 and C253. The structural variation between polymorphisms is mainly located in the middle region and C-terminal region [10, 27, 57]. The high number of β-strands results in a highly stable fibril core, which could potentially be irreversible once formed, as suggested by Jiang et al. [27]. The S120 is buried deep within the fibril core, leaving no space for additional amino acids. Therefore, fibrillization may only occur when TMEM106B is cleaved at residue 119.

Based on *TMEM106B* genotypes, singlet I can be formed by either the T185 or S185 isoform (encoded by the risk and protective *TMEM106B* haplotypes, respectively) considering it is present in TT, TS, and SS individuals, while singlet II is only observed in TS and SS individuals. This leaves the possibility that the packing of the singlet II fibril leaves insufficient space for a threonine residue and can only be formed by the S185 isoform, as suggested by Schweighauser et al. [57].

Though Schweighauser et al. [57] report all polymorphisms to be capable of forming doublets, only the complete cryo-EM structure of two polymorphs of doublets comprising two protofilaments of singlet I have been fully resolved. In doublet I, the two singlets are arranged with a twofold symmetry centered around the positively charged residues K178 and R180 with an unidentified non-proteinaceous anionic cofactor in the middle, facilitating doublet formation [10, 27, 57]. Alternatively, doublet II is centered around Glu206, Met207, Tyr209, and Tyr211 [27].

Of note, the normal C-terminal portion of TMEM106B comprises AA118-274, meaning that the last 20 amino acids are not present within the fibril core. It is not clear whether the last amino acids are cleaved off, or alternatively reside outside of the fibril. While it was proposed to be potentially present as a fuzzy coat by Jiang et al. [27], similar to what has been observed for Tau [60], α-synuclein [58], TDP-43 [2], and Aβ[31], Schweighauser et al. described TMEM106B fibrils as a fibril that seemed to lack a fuzzy coat based on the electron micrographs [57].

### Updated view on TMEM106B biology

#### Proteolytic processing

**Fig. 4:**
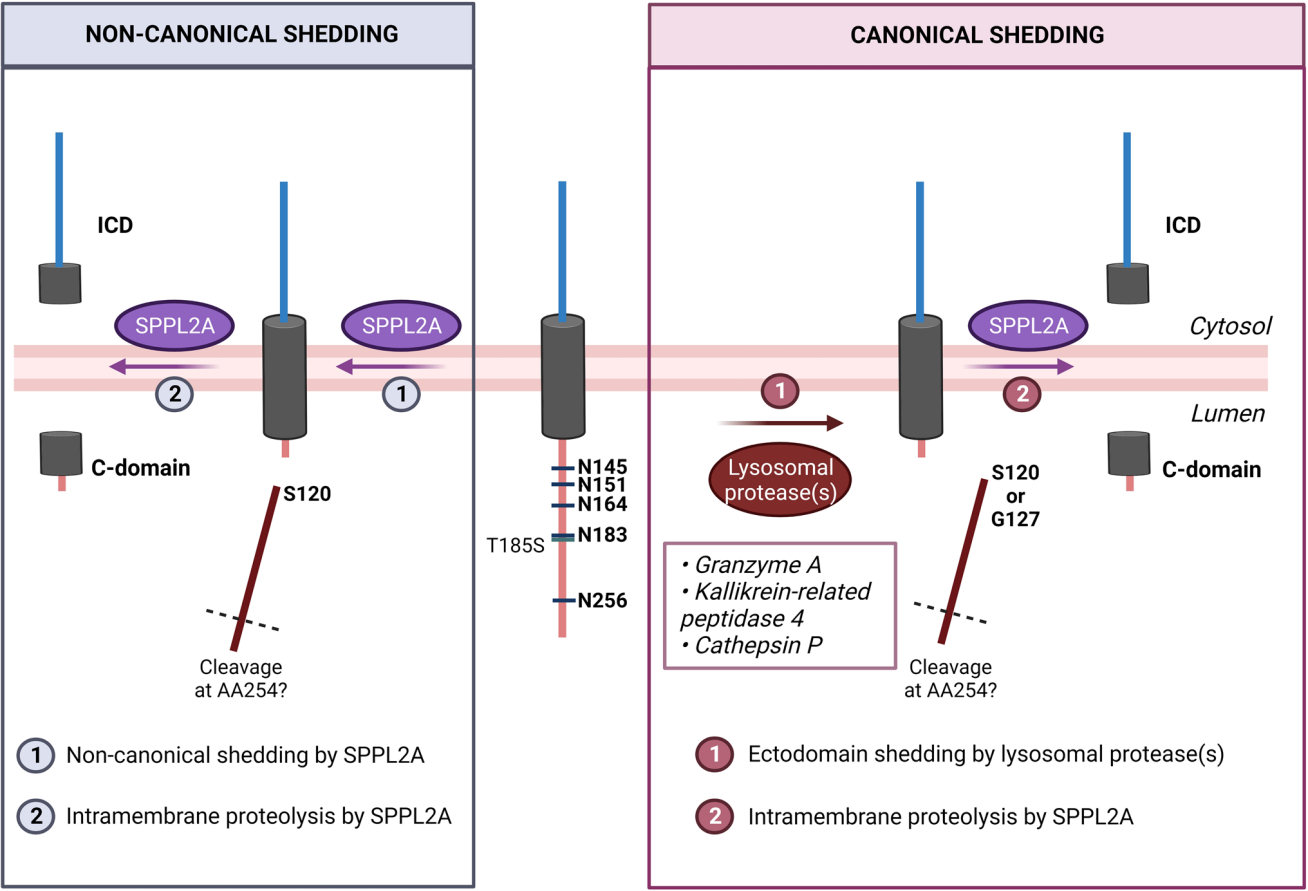
An updated view on TMEM106B biology and processing at the endolysosomal membrane. Prior to intramembrane proteolysis by SPPL2a, the C-terminal domain of TMEM106B may be released by canonical shedding through a lysosomal protease. Chang et al. proposed three novel candidate enzymes, which are indicated in the pink box. Conversely, Schweighauser et al. suggest that this region may not be accessible to lysosomal proteases. Instead, TMEM106B’s C-terminal domain may be released through non-canonical shedding. This may potentially also occur by SPPL2a, similar to non-canonical shedding of TNF-α [62]

Considering that the proteolytic cleavage of TMEM106B is a critical event to form fibrils, further studies on factors modulating proteolytic processing such as the involved protease(s) and physiological environment will be crucial. While Chang et al. queried the neighboring sequence to identify potential proteases (granzyme A, kallikrein-related peptidase 4, cathepsin P) [10], Schweighauser et al. proposed that, in the native structure, the globular domain is located too close to the lysosomal membrane. They argue that the lack of a flexible linker and the hydrophobic surface patch at the end of the domain make it highly unlikely that S120 is accessible to a lysosomal protease. Rather, the authors propose the existence of a non-canonical shedding pathway [57]. The C-terminal domain shedding of TMEM106B may therefore occur through canonical shedding of the C-terminal domain of TMEM106B by an unidentified lysosomal protease at position G127, as described before [6], and/or at position S120. Alternatively, the S120 C-terminal domain is released through non-canonical shedding of TMEM106B. Interestingly, the existence of non-canonical shedding by SPPL2A has recently been reported for TNF-α [62]. After shedding the C-terminal domain, SPPL2A may further cleave TMEM106B within the membrane (Fig. 4).

It is interesting to speculate that canonical shedding by a lysosomal protease indeed occurs at position G127, as reported in [6], rendering C-terminal fragments generated by this process incapable of forming fibrils. Canonical shedding at position G127 and non-canonical shedding at position S120 may both occur in physiological circumstances with different efficiencies. In disease, the shedding process might be skewed more toward the non-canonical way, for example, due to lysosomal dysfunction and/or reduced activity of the lysosomal protease responsible for canonical shedding. This would increase the amount of protein that is processed into the fibrillogenic C-terminal peptide (S120) to maintain the amount of functional full-length protein present on the lysosomal membrane.

#### Fibril formation

**Fig. 5:**
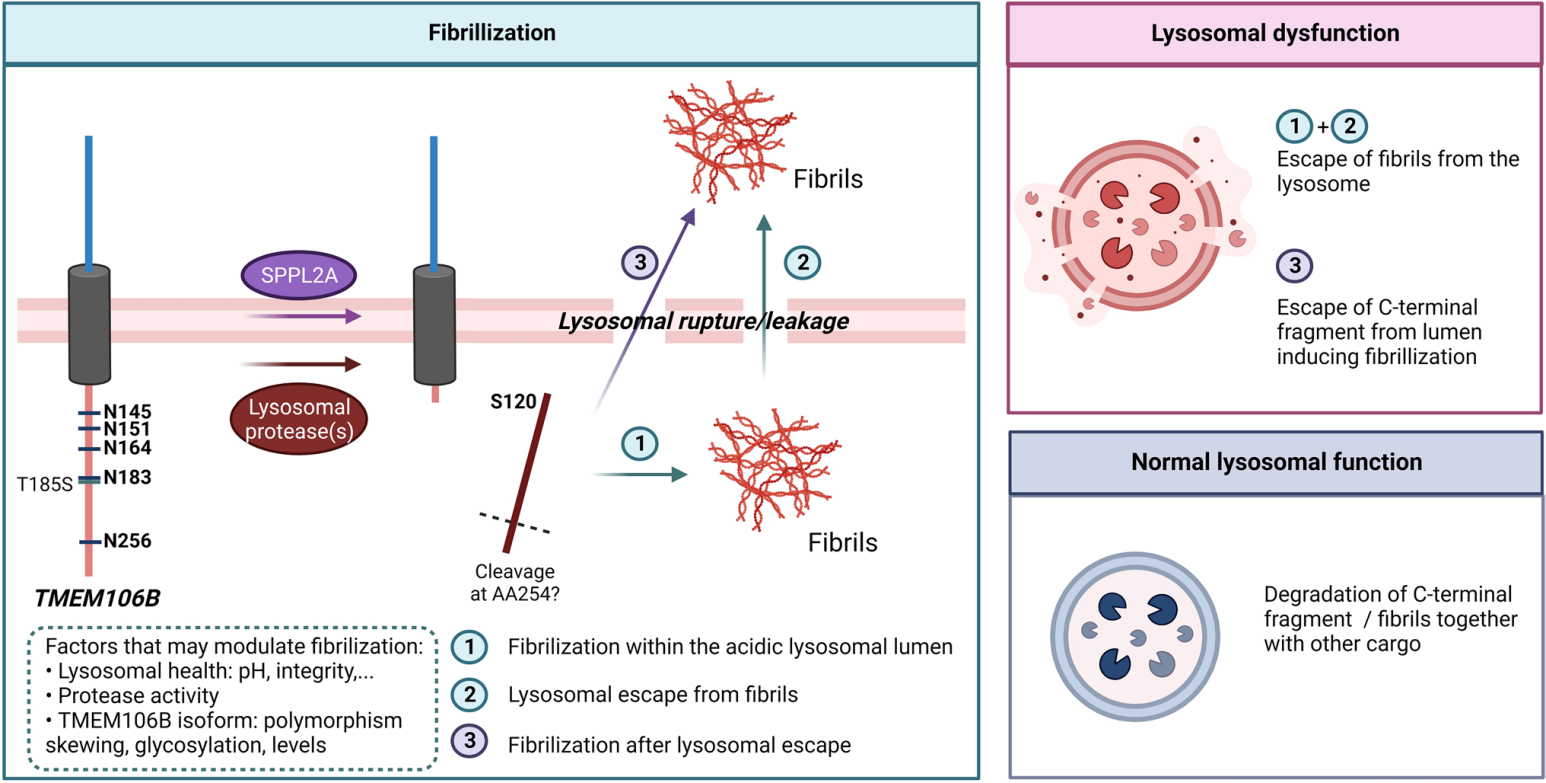
Fibril formation of TMEM106B. After shedding the C-terminal domain, fibrils may form within the acidic lysosomal lumen. Lysosomal leakage or rupture, as a consequence of lysosomal dysfunction, may allow them to escape to the cytosol. Alternatively, fibrils may form within the cytosol once the C-terminal domain escaped the lysosomal lumen. In normal circumstances, TMEM106B’s C-terminal domain or fibrils will be degraded along with other lysosomal content. Factors that may modulate fibrillization are related to lysosomal function (pH, protease activity, lysosomal integrity, etc.) or as a result of the TMEM106B isoforms (polymorphism skewing, glycosylation, levels)

Due to the lack of detailed intracellular stainings such as co-stainings with organelle markers, it is not clear whether the fibrils form within the lysosomal lumen or whether the C-terminal domain first needs to escape the lumen. This leaves two possibilities (Fig. 5): (I) the fibrils form within the lumen and potentially later escape the lysosome, or (II) the fibrils can only form within the cytosol once the C-terminal domain is released from the lumen. In this regard, Jiang et al. remarked that the presence of negatively charged amino acids directed at each other suggests that the fibrils can only form in an acidic environment, where the negative charges are attenuated, as would be the case in the acidic lumen of the lysosome [27].

Lysosomal health will be an important factor mediating the fibrillar burden as TMEM106B is primarily localized and processed within the lysosome. In addition, the lysosome will likely also be responsible for the degradation of the C-terminal fragment and/or TMEM106B fibrils. Lysosomal dysfunction may alter fibrillization by affecting pH, and protease activity, or may lead to an increment in fibrils within the lumen due to impaired degradative function. Impaired lysosomes may also release the fibrils within the cytosol in case of lysosomal rupture, or within the extracellular space due to lysosomal exocytosis. Interestingly, lysosomal exocytosis is increased when TMEM106B levels are elevated [21, 33], which might induce spreading of TMEM106B fibrils to neighboring cells.

#### TMEM106B haplotypes

The discovery of TMEM106B fibrils revives the question of the functional effect of the *TMEM106B* risk/protective haplotype. Considering all three combinations of haplotypes (TT, TS, SS) were represented in the study population, it is apparent that both the T185 isoform and the S185 isoform can form fibrils. Because it is not known whether the T185S variant located on the haplotype is responsible for the effect, we describe both the potential contribution of the T185 and S185 isoform as well as the described increase in expression levels. While in theory we can distinguish these hypotheses as separate possibilities, in all likelihood these mechanisms converge and are contributing to the process together.

(I) Polymorphism skewing (p.T185S): as the current cryo-EM resolution was not able to distinguish a serine from a threonine, it is not known whether the isoforms are equally represented in the fibrils.
(II) Glycosylation status (p.T185S): all fibril polymorphisms are stated to be fully glycosylated and Schweighauser et al. reported residues G177-N183 to form a conserved conformation in all folds. It was shown that N–X–T is glycosylated more efficiently than N–X–S [7], which raises the possibility that the p.T185S variant may alter TMEM106B fibrillization by altering the glycosylation status at position N183 and therefore fibril formation. After all, post-translational modifications are known to affect fibrillization of other aggregating proteins [1, 68].
(III) Fibril burden (Levels/p.T185S): alternatively, it may be the described increase in TMEM106B protein levels that may directly modulate fibrillar burden which might underlie a potential modifying effect of the risk/protective haplotype, either due to expression changes (chromatin looping) [22] or decreased stability of the S185 isoform [46].
(IV) A final possibility is that the proteolytic processing of the S185 isoform is different from that of the T185 isoform due to altered binding affinity or substrate recognition.

### Disease implications

#### TMEM106B in brain aging and neuronal health

The observation of TMEM106B fibrils across multiple neurodegenerative disorders is not surprising given the extensive repertoire of disease associations previously reported for TMEM106B. It is nevertheless intriguing that all cryo-EM reports draw markedly different conclusions on the role of TMEM106B fibrils in the process of neurodegeneration. While Jiang et al. define the fibrils of FTLD-TDP patients as being solely constituted of TMEM106B with a possible central role in disease [27], Schweighauser et al. claim that TMEM106B fibrils form in an age-dependent manner in the brain without a mechanistic connection to disease [57]. It is important to note that neurodegenerative disease hallmarks such as amyloid plaques or TDP-43 aggregates are also observed in apparently healthy individuals, though to a more limited extent [53, 54]. The mere presence of TMEM106B fibrils in neurologically normal individuals is therefore not sufficient to exclude a pathogenic effect of the fibrils and it may thus be premature to classify the fibrils as generic byproducts similar to lipofuscin. Rather, the age-dependent accumulation of TMEM106B fibrils also in healthy controls may potentially underlie the age-dependent association of *TMEM106B* risk haplotype with differential aging and neuronal proportion [38, 54]. The post-mitotic nature of neurons makes them highly vulnerable to the stressors accompanied by the aging process such as oxidative stress and buildup of damaged proteins [32, 40, 44]. These stressors put a strain on the endolysosomal system, which could result in the accumulation of TMEM106B fibrils even in the absence of disease which might be neurotoxic in a high enough concentration. Consequently, fibrillization of TMEM106B by itself may contribute to neuronal loss. Additionally, the presence of TMEM106B fibrils might diminish the brain’s resilience against neurodegeneration by influencing neuronal health.

Given the large number of genetic associations of the *TMEM106B* haplotypes to neurodegenerative disorders and general brain aging, it seems unlikely that the fibrils are benign. Yet, TMEM106B has only been identified as a risk factor for neurodegenerative disorders and a potentially toxic accumulation of a protein only associated with disease risk has not been described before. This leaves the possibility that disease-causing mutations in TMEM106B still need to be identified or that the risk- and disease-modifying effect of the TMEM106B is unrelated to TMEM106B fibrils and potentially occurs prior to their formation. TMEM106B regulates several aspects of lysosomal functioning and alterations in TMEM106B’s biology and function, be it TMEM106B expression levels or a functional consequence of p.T185S, may therefore underlie the disease-modifying effect regardless of fibril formation. Considering TMEM106B C-terminal fragments are likely degraded within the lysosome under normal circumstances, it should be noted that the formation and presence of TMEM106B fibrils may be representative of lysosomal dysfunction in general and not necessarily actively contribute to neurotoxicity or pathology. Regardless, the findings of TMEM106B amyloid fibrils in disease and normal brains bring a new dimension to the involvement of TMEM106B in brain health.

#### TMEM106B in proteinopathies: lysosomal dysfunction as the central hub

Given that neurodegeneration and brain diseases are complex disorders with several contributing factors, there might not be one answer to explain the role of TMEM106B fibrils in all implicated disorders. The contribution of TMEM106B fibrils to disease may depend on the affected brain region as well as the inflicted cell types or underlying pathomechanisms. It is also possible that in some neurodegenerative disorders, TMEM106B fibrils will be active drivers of the pathogenic process, while in other disorders TMEM106B fibrils might act as secondary bystanders that may promote or accelerate disease pathology and aggravate disease phenotype. A variable contribution of TMEM106B fibrils to disease pathogenesis is also supported by the nature of the observed genetic associations of TMEM106B with disease. For example, in FTLD-TDP caused by *GRN* mutations, the *TMEM106B* risk haplotype modifies disease risk to such an extent that people who are homozygous for the protective *TMEM106B* haplotype may remain lifelong symptom free, whereas in Parkinson’s disease and ALS the *TMEM106B* haplotype association is restricted to an effect on the degree of cognitive decline [36, 51, 67]. However, it remains to be determined whether TMEM106B fibrils are more frequently associated with certain proteinopathies, which will require detailed unbiased studies of large cohorts.

**Fig. 6:**
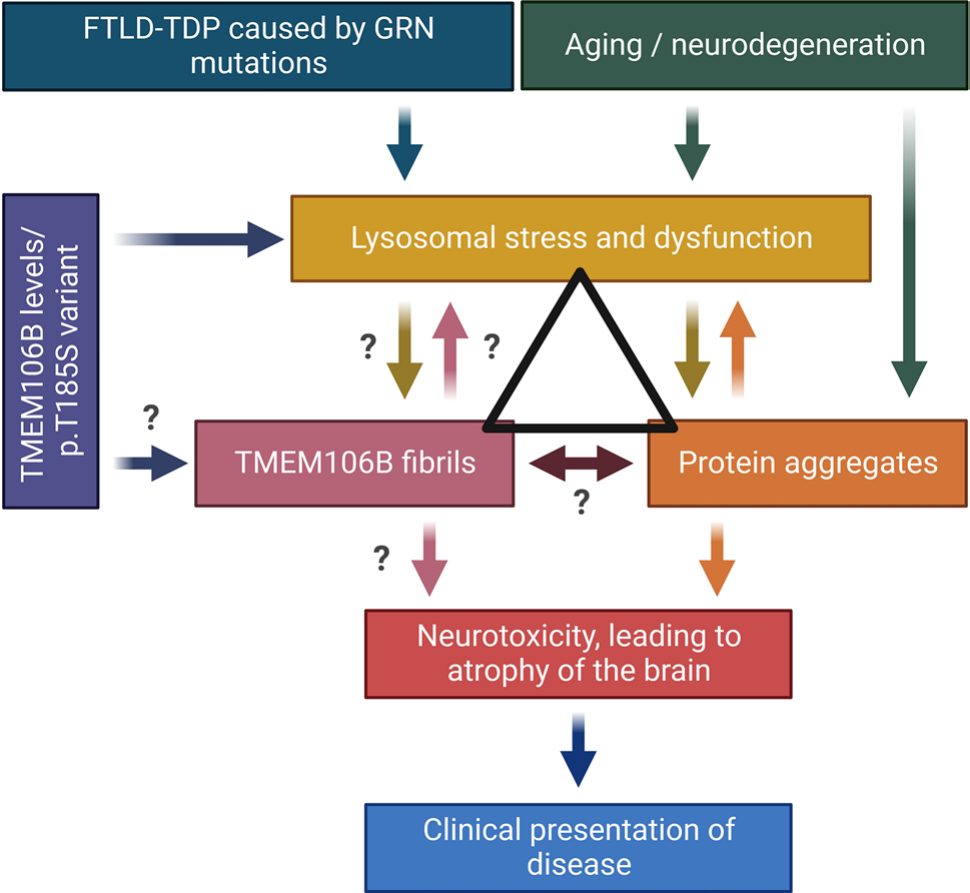
Potential pathomechanism and disease contribution of TMEM106B fibrils*.* Lysosomal dysfunction is represented as central hub within the disease mechanism. The contribution of TMEM106B fibrils to disease might depend on the importance of the lysosome within the specific pathomechanism. The p.T185S variant might modulate disease by affecting lysosomal health through modulation of TMEM106B levels or by affecting TMEM106B fibrillization. TMEM106B fibrils may actively drive the disease mechanism in FTLD-*GRN*, as PGRN and TMEM106B functionally converge within the lysosome and are thus affected early in the disease mechanism. In other disorders or during aging, the lysosome is only affected in a later stage and TMEM106B fibrils may play a more secondary role, potentially promoting or accelerating disease pathology

While it is not clear where exactly the fibrils reside, within lysosomes or in the cytosol, TMEM106B is localized in lysosomes and is proteolytically processed at the lysosomal membrane defining the lysosome as a central hub. Lysosomes have gradually been recognized as important players in neurodegenerative disorders [3, 26, 44, 55, 75], thus it is plausible that the contribution of TMEM106B fibrils to disease might depend on the importance of the lysosome within the specific pathomechanism (Fig. 6).

#### FTLD-TDP caused by *GRN *mutations: TMEM106B fibrils as primary disease protein

The exceptionally strong disease-modifying effect in *GRN* mutation carriers likely occurs within the endolysosomal system as PGRN and TMEM106B are both important players in maintaining lysosomal health. In fact, homozygous mutations in *GRN* have been shown to cause a lysosomal storage disorder, neuroid lipofuscinosis (NCL) [61]. PGRN is cleaved within the lysosome into functional granulins and affects several aspects of lysosomal function, including the activity of lysosomal enzymes such as cathepsin D [49, 75]. Mutations in *GRN* result in dysfunctional lysosomes and it is tempting to speculate that loss of PGRN/granulins will directly affect TMEM106B processing or fibril formation by modulating lysosomal health.

Interestingly, knock-out of *Tmem106b* worsens disease pathology in *Grn-/-* mouse models and induces the accumulation of phosphorylated TDP-43 in an age-dependent manner [20, 69, 74]. A recent report also showed TMEM106B knock-out to result in an increase in TDP-43 cytoplasmic aggregates in a cellular model for TDP-43 proteinopathy [43]. These findings together with the identification of TMEM106B fibrils in FTLD-TDP highlight a direct, seemingly complex relationship between TMEM106B and TDP-43. At a minimum, it confirms the importance of the lysosome in the disease mechanism. Two of the main TDP-43 degradation pathways are mediated by lysosomes, e.g., chaperone-mediated autophagy (CMA) occurring in the lysosomes and the autophagosome–lysosome pathway itself [55], and TMEM106B knock-out disrupts normal lysosomal functioning [6, 63]. Second, it suggests that the loss of function of TMEM106B, potentially caused by TMEM106B fibrillization, may contribute to TDP-43 mislocalization and aggregation.

Of note, TDP-43 knock-out in its turn also affects lysosomal biology and induces the abnormal accumulation of lysosomes in the perinuclear region, a decrease in cathepsin L and PGRN to the lysosome, abnormal cathepsin B processing, and secretion of undigested proteins from the lysosome [35, 39, 55]. This means that the mislocalization and/or accumulation of TDP-43 may further induce the formation of TMEM106B fibrils by disrupting lysosomal function, which will further compromise the degradation of the cleaved C-terminal domain of TMEM106B as well as clearance of TDP-43 aggregates which could set in motion a feedback loop.

#### Other proteinopathies: TMEM106B fibrils as secondary disease protein

Alternatively, protein aggregation and subsequent lysosomal dysfunction, caused by other environmental or genetic factors, may be upstream of the formation of TMEM106B fibrils which could potentially contribute to or aggravate disease pathology. A contribution of TMEM106B fibrils to disease may also present itself more as a disease modifier where the formation of TMEM106B fibrils contributes to disease manifestation, for example modifying cognitive decline in Parkinson’s disease and ALS [36, 67]. A potential effect of TMEM106B in contributing to the formation of other aggregating proteins may be supported in TDP-43 proteinopathies by the observation that the *TMEM106B* risk variants have been associated with increased TDP-43 aggregates in neuropathology-based association studies of apparently healthy older individuals [15, 73] and the observation that having the protective allele reduced TDP-43 burden in C9orf72 expansion carriers [4].

## Conclusion and future directions

The identification of TMEM106B amyloid fibrils will undoubtedly transform research on TMEM106B and its involvement in neurodegeneration, but simultaneously raises a lot of new questions. First, the pathogenicity and disease contribution of the fibrils need to be determined, especially since fibrils were also observed in aged individuals without neurodegenerative diseases. Second, detailed studies on the contribution of the haplotype are required. Regardless of the precise mechanism, it is possible that the *TMEM106B* risk haplotype either indirectly affects the fibrillar burden by modulating TMEM106B levels or processing, or directly by affecting glycosylation at position N183. Third, it will be important to elucidate the precise mechanism, the involved proteases, and cellular conditions required for TMEM106B fibrillization as well as to determine the precise location of the fibrils and the identity of the cofactor within the doublets. Finally, since TMEM106B fibrils were found in neurodegenerative disorders that are typically characterized by aggregation of other proteins, such as TDP-43, it will be important to determine whether there could be a synergistic effect on the burden of other aggregated proteins.

However, the most urgent challenge the field currently encounters is the need to generate suitable antibodies and other tools to be able to study TMEM106B and its fibrillization mechanism. Antibodies will be necessary to perform correlation studies in large patient cohorts to investigate the contribution of TMEM106B pathology to disease as well as to be able to study TMEM106B biology such as its proteolytic processing. Depending on the outcome of these vital studies, TMEM106B fibrils may be pursued as a biomarker or even as a therapeutic target. Yet, this might not be straightforward considering the tight regulation of TMEM106B levels within the cell, with both knock-down and overexpression of TMEM106B resulting in lysosomal dysfunction. This means that therapeutic interventions targeting TMEM106B will need to be precisely monitored and requires detailed knowledge of TMEM106B fibril formation, as well as its normal function.
